# Bifurcation matching for consistent cerebral vessel labeling in CTA of stroke patients

**DOI:** 10.1007/s11548-022-02750-9

**Published:** 2022-10-01

**Authors:** Leonhard Rist, Oliver Taubmann, Florian Thamm, Hendrik Ditt, Michael Sühling, Andreas Maier

**Affiliations:** 1 Pattern Recognition Lab, FAU Erlangen-Nuernberg, Martensstrasse 3, Erlangen, 90156 Germany; 2 Siemens Healthineers, Siemensstr. 3, Forchheim, 91301 Germany

**Keywords:** Vessel labeling, Vessel identification, CTA, Stroke

## Abstract

**Purpose:**

Vessel labeling is a prerequisite for comparing cerebral vasculature across patients, e.g., for straightened vessel examination or for localization. Extracting vessels from computed tomography angiography scans may come with a trade-off in segmentation accuracy. Vessels might be neglected or artificially created, increasing the difficulty of labeling. Related work mainly focuses on magnetic resonance angiography without stroke and uses trainable approaches requiring costly labels.

**Methods:**

We present a robust method to identify major arteries and bifurcations in cerebrovascular models generated from existing segmentations. To localize bifurcations of the Circle of Willis, candidate paths for the adjacent vessels of interest are identified using registered landmarks. From those paths, the optimal ones are extracted by recursively maximizing an objective function for all adjacent vessels starting from a bifurcation to avoid erroneous paths and compensate for stroke.

**Results:**

In 100 CTA stroke data sets for evaluation, 6 bifurcation locations are placed correctly in 85% of cases; 92.5% when allowing a margin of 5 mm. On average, 14 vessels of interest are found in 90% of the cases and traced correctly end-to-end in 73.5%. The baseline achieves similar detection rates but only 35.5% of the arteries are traced in full.

**Conclusion:**

Formulating the vessel labeling process as a maximization task for bifurcation matching can vastly improve accurate vessel tracing. The proposed algorithm only uses simple features and does not require expensive training data.

## Introduction

**Fig. 1:**
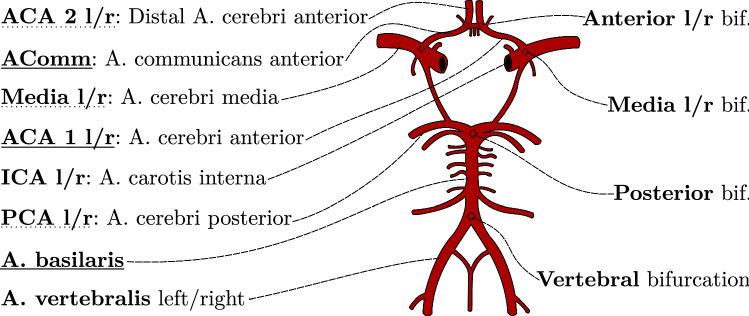
Normal configuration of the CoW. Analyzed arteries are on the left (inner underlined, outgoing dotted), bifurcations of interest are on the right

The Circle of Willis (CoW) is a central structure in the human cerebral vasculature which bundles the incoming blood and redistributes it to all brain areas (configuration in Fig. 1). A thrombus blocking a vessel limits the oxygen supply to the brain, causing an ischemic stroke which is a leading cause of disability in adults [1]. Computed Tomography Angiography (CTA) is the modality of choice to visualize vessels quickly and accurately. Since the location of an occlusion is crucial for determining therapy, anatomical labeling of the major arteries is highly important. It facilitates navigation and serves as a prerequisite for comparing patients using vessel straightening [2]. For such tasks, the complete vessel path, as opposed to the rough location only, is required. An automatization can be helpful in the time-critical acute stroke diagnosis.

High-quality segmentation of fine cerebral vessel structures is often achieved by Digital Subtraction Angiography (DSA; requires two CT scans) or magnetic resonance angiography (MRA), which may be infeasible due to time constraints in case of suspected stroke, hence using such data would not fit our requirements [3]. Vessel segmentation in CTA scans is highly challenging. As manual annotation of vessels is time-consuming, supervised machine learning methods may not always be an option. However, there also exist non-trainable segmentation methods such as the region-growing approach VirtualDSA++ by Thamm et al. [4] which is used representative in this work. Due to factors, such as noise or bones, segmentations obtained from CTA may be of lower quality than ones based on MRA or DSA. As a consequence, cerebrovascular models from CTA segmentations can be expected to have missing smaller structures or to include artificial connections, leading to problems in the labeling.

### Related work

Most published cerebral vessel labeling algorithms are designed on MRA data and possibly unaware of such complications. Bogunovic et al. [5] use maximum a posteriori estimation after learning bifurcation features on a graph and focus on robustness against anatomical variations without explicitly considering stroke. Also relying on a small MRA data set, Bilgel et al. [6] utilize belief propagation together with a Random Forest classifier. In contrast, Dunås et al. [7] label MRA segmentations by locating the longest segments within a graph generated from a custom atlas which may be problematic for the noisier CTA segmentations. Robben et al. [8] tackle the problem earlier by first constructing an overcomplete graph which is assumed to contain the correct segmentation and optimize for the graph fulfilling the desired labeling.

Fewer labeling approaches in literature focus on CT data. Ghanvanti et al. [9] use micro-CT of mice in their anatomical labeling, following the ideas of Bilgel and Bogunovic by formulating a stochastic relaxation problem. Yao et al. [10] proposed a graph convolutional approach to improve their segmentation and then perform semantic labeling for head and neck vessels on CTA data sets. Thriving for a simplistic method, Shen et al. [2] register end and start artery key points in the graph to label them via distance measures. Afterward, they use a shortest-path algorithm to define the pathways and employ a deep learning approach for the difficult area of the ACA 2 segment. However, they state no details regarding their CTA segmentation approach and do not address the problems such as artificial loops when using a shortest-path algorithm.

**Fig. 2:**
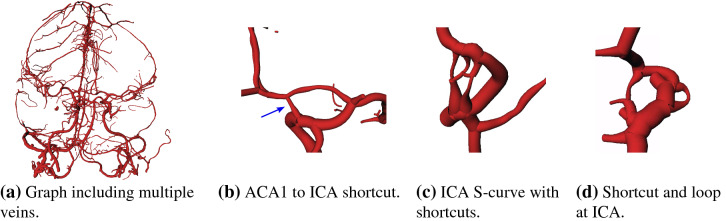
Examples of vessel graphs and segmentation artifacts

### Contribution

None of the methods above consider stroke and were often designed on higher-quality MRA data while we evaluate on practically relevant CTA data. Furthermore, virtually all methods require training data in some form. To achieve automated vessel labeling for ischemic stroke applications without expensive annotations we propose a method combining marker registration and graph-based recursion on bifurcation level. This algorithm works on cerebrovascular graph models created from a CTA vessel segmentation.

To obtain consistent labeling, we identify bifurcations by determining and subsequently fusing optimal sub-graphs. First, vessel scores are computed using a cost function incorporating the smoothness of the vessel course, length, radius and distance to atlas key points. From each possible bifurcation node, all paths are traced and rated recursively, starting at its adjacent edges to determine the best candidate. This global approach overcomes the issues of artificial multiple paths in the vessel tree which may mislead locally operating methods and improves robustness against occlusions. We compare our method to a baseline based on registration of atlas landmarks to demonstrate the benefit of a bifurcation- and graph-based solution. Results are presented for the accuracy in identifying 6 relevant bifurcations as well as for the detection and path tracking accuracy of 14 major arteries, which are all displayed in Fig. 1.

## Method

### Data

On a total of 105 thin-slice CTA data sets of stroke patients (5 patients for development; 100 for evaluation), vessel segmentation was performed with the VirtualDSA++ [4] algorithm to create cerebrovascular models. The segmentations of 66 cases exhibit missing segments in the arteries of Fig. 1 mostly due to stroke but occasionally also due to segmentation inaccuracies which are treated equally in this work. Each patient is represented as a graph *G*(*V*, *E*) where the nodes *V* describe the bifurcations and endpoints of vessels and the edges *E* describe the interconnecting vessel segments. Each node *v* holds its position, each edge *e* contains a sequence of points and radii *r* to define the vessel centerline. The number of nodes and edges varies substantially as a consequence of unequal imaging phases or varying amounts of contrast agent, resulting sometimes in large graphs as shown in Fig. 2a. Segmentation artifacts are shown in Fig. 2b–d, including shortcuts or loops, which cause problems with shortest-path approaches or naïve landmark registration. These errors are also expected to occur in similar (region-based) segmentation algorithms.

### Algorithm

#### Registration

**Fig. 3:**
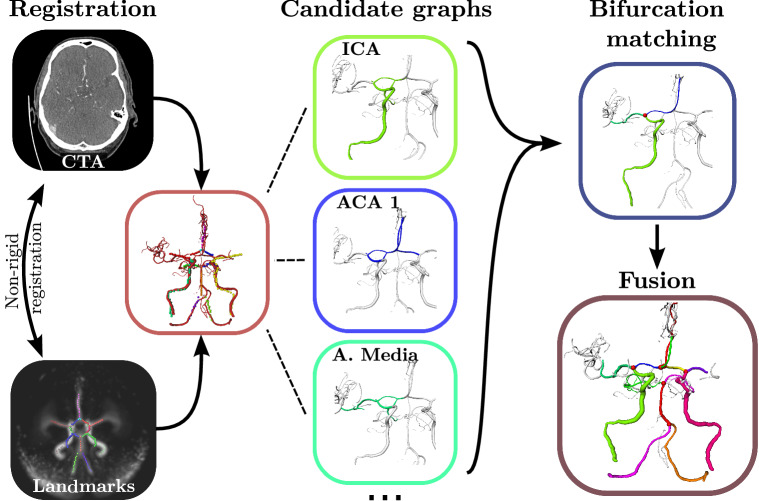
Algorithm pipeline using the example of the Media bifurcation creation

A brain atlas [11] was used in combination with a vessel atlas [12] (containing non-vessel-specific probabilities) to draw landmarks for the arteries of interest, cf. Fig. 3. By non-rigidly registering the brain atlas to the patient volume, these landmarks can be transformed accordingly. The brain vasculature is rich in variations and may not match the soft-tissue-based registration perfectly. Therefore, we also rigidly align the landmarks to the segmented centerlines using the Iterative Closest Point (ICP) algorithm [13].

#### Candidate graphs

The next goal is to create candidate sub-graphs for each artery based on distance measurements. These measurements will be utilized below to identify bifurcations and to eliminate unlikely vessel segments to limit the search space. A distance score $d_a(e)$ for the artery *a* and vessel segment *e* holding *N* centerline points with positions $\mathbf {x}_n$, $n\in \{1,\ldots ,N\}$ is computed by using a set of *M* landmarks $\mathbf {x}^{(a)}_m$, $m\in \{1,\ldots ,M\}$:

1 $$\begin{aligned}&d_a(e) = \frac{1}{N}\sum _{n=0}^N s_n + \sqrt{\frac{1}{N}\sum _{n=0}^N (s_n - \min _{0<i\le N}(s_i))^2}, \nonumber \\&\text {with}\, s_n = \min _{0<m\le M}(\Vert \mathbf {x}_n-\mathbf {x}_m^{(a)}\Vert _2). \end{aligned}$$

For each artery landmark set, the Euclidean distance from all centerline points per segment/edge is computed to the closest landmark and averaged over the segment to calculate a distance value. Points that match one of the two landmarks at the ends of the artery are omitted. The distance value is extended by a spread computation to punish segments perpendicular to our marker array. For this purpose, we modify the standard deviation by utilizing the minimum instead of the mean to detect whether a segment is parallel or perpendicular to our landmark groups. All segments for which $d_a(e)$ is smaller than a threshold of 2 cm are combined to a candidate sub-graph per artery, see Fig. 3. This empirical value limits the search space for the next step while serving as an upper bound to compensate for registration errors and positional variations. It is also consistent with the evaluation range for outgoing vessels, see section “Evaluation”.

#### Bifurcation matching

To achieve consistent labeling the endpoints of the three vessels belonging to a bifurcation must be equal. Our goal is to find the best matching node for each bifurcation first instead of directly matching the most likely arteries in terms of distance to landmarks. Deformations of the vascular tree can thus be compensated and the labeled vessel transitions are always consistent. The second important concept is the consideration of all paths artery starting from this node. Since vessel edges can have different lengths over all patients it is insufficient to only consider local neighborhoods.

We design a cost function *q* to create scores for whole vessel paths with regard to specific arteries and evaluate it for all possible outgoing vessel paths for one candidate node. The scores over all three arteries of a bifurcation are then averaged and the node with the best score is assigned. Therefore, even with missing vessels, e.g., due to stroke, it is still possible to match a bifurcation if the rest of the vessels match well enough to create a high score. In defining the cost for a vessel path, we consider the vessel length *l*, the distance *d* to the landmark points, the radius *r* as well as the angle $\alpha$ between adjacent edges.

The length is important as major arteries are expected to be long. Conversely, a larger distance *d* should lead to a lower score. Furthermore, the vessel path should be smooth, meaning angles between adjacent edges are expected to be close to $\alpha = 180^{\circ }$. The angle between two edges is linearly transformed into a weight *w* in the range [0.2; 1.0] by utilizing the dot product.

This weight should affect all subsequent edge scores, so its cumulative product will be used in combination with the value itself. The influence of the radius should be rather low with $\delta =0.1$. The scoring function $c_a(e,\mathbf {w})$ for one edge *e* and a set of *N* weights $\mathbf {w}$ is defined as

2 $$\begin{aligned} c_a(e,\mathbf {w}) = \frac{(w_N + \prod _i^N w_i)}{2} \cdot \frac{l_e}{d_a(e)} \cdot (1 + \delta \cdot r_e). \end{aligned}$$

Next, we define the function *q* to recursively compute the maximum score for a vessel path starting at a node *v* and edge *e*. To navigate through the candidate edge set $E_a$ we define the neighboring edge set of an edge *e* without the edges at one of its adjacent nodes *v* as $n_e(e,v)$. The corresponding node connecting *e* with $n_e(e,v)$, i.e., its other adjacent node, is defined as $n_v(e,v)$:

3 $$\begin{aligned}&q_a(e,v,E_a,\mathbf {w}) = {\left\{ \begin{array}{ll} c_a(e,\mathbf {w}) &{} n_e(e,v) = \emptyset ,\\ c_a(e,\mathbf {w}) &{} n_e(e,v) \not \in E_a,\\ c_a(e,\mathbf {w}) + \max \limits _{f \in n_e(e,v)}(q_a(f,\tilde{v},E_a\backslash \{e\},\tilde{\mathbf {w}})) &{} \text {otherwise,}\\ \end{array}\right. } \nonumber \\&\quad \text {with } \tilde{v} = n_v(e,v) \text { and } \tilde{\mathbf {w}} = \mathbf {w} \cup \{w(e,f)\}. \end{aligned}$$

The first recursive function call at a bifurcation candidate node *b* and its adjacent edge $e_1$ for the artery $a_1$ is executed using $q_{a_1}(e_1,b,G_{a_1},[1]).$

The best bifurcation points are now calculated by applying above functions on all adjacent edges of a node for all three expected arteries (see example of ICA, ACA 1 and A. media for the Media-L bifurcation in Fig. 3). Hence, for a node with 3 neighbors, 6 different configurations are calculated and the three values of $q_a$ for each artery are averaged, respectively. Using the maximum of these values over all possible bifurcation nodes, we receive the optimal node. Possible bifurcations nodes are within a 1.5 cm distance to the endpoint landmarks of their arteries. This process is repeated for all bifurcations until each has been assigned to a graph node, together with three optimal pathways for its adjacent arteries which form a bifurcation sub-graph. The resulting six bifurcation graphs may, however, overlap and thus need to be fused for a complete labeling. Note that it is possible that vessels or bifurcations are not matched at all, e.g., in case of strokes, segmentation errors or anatomical variants.

#### Fusion

The bifurcation sub-graphs contain the inner arteries AComm, ACA 1 and the A. basilaris twice while the rest are outer arteries and only included once. The inner arteries of the respective candidate graphs need to be merged according to their overlap. When that intersection is $\emptyset$, the shortest path between the endpoints of the candidate edges is chosen. Outer edges are included as computed by the bifurcation optimization. Since their length varies and may exceed the provided landmarks, tracing is applied at their ends by repeatedly including the next edge with the largest transition angle above $120^{\circ }$. Furthermore, the two Anterior bifurcations might be incorrectly matched onto the same node due to the similarity of the two ACA 2s. However, this double assignment would indicate the presence of only a single ACA 2 which is exhibited in some CoW variants. To distinguish these cases, the second best node for the bifurcation with the lower score (e.g., left) is checked. If its score is within an empirically determined range of 80% of the best score (e.g., right), the bifurcation is reassigned to this second-best location as it can be assumed that two ACA 2s exist. Special handling of this area is also done e.g., by Shen et al. [2]; whereby the anterior arteries are in general not relevant for stroke.

**Table 1:** Result categorization classes and subclasses used for evaluation

Correct	TP*	Complete	Vessel is labeled completely correct
	TP−	Underestimated	Main part of the vessel found, not complete
	TP+	Overestimated	Complete vessel found plus additional part
	TP±	Misestimated	Both over- and underestimated
	$\text {TP}_{\textsc {SUM}}$	Detected	Sum of all above
	TN	Dismissed	Vessel not detected (vessel not present)
False	FN	Not detected	Vessel not found (vessel present)
	FP+	Misplaced	Incorrect vessel labeled (vessel present)
	FP−	Wrongly detected	Incorrect vessel labeled (vessel not present)

**Table 2:** Bifurcation accuracies

Labeling	Vertebral (%)	Posterior (%)	Media L (%)	Media R (%)	ACA L (%)	ACA R (%)	Mean (%)
Correct	91	94	83	81	83	77	84.8
$\le$ 5 mm	91	95	93	93	93	90	92.5
False	9	5	7	7	7	10	7.5

Each value represents the average rate over all 100 patients. “$\le$ 5 mm” also includes bifurcations within a 5 mm distance

### Evaluation

#### Baseline

We use a naïve distance-driven baseline approach to show the advantage of using the proposed recursive scheme over a simple matching. The edges are sorted in descending order by their radius and are processed successively. The distance from the edge to the landmark sets is computed analogously to the distance computation for the creation of the candidate graphs, including the registration, ICP matching and distance metric of Eq. 1. An edge is assigned to the closest artery landmark set if the distance metric is below 2 cm (as in the proposed algorithm) and if one of their neighboring edges has already been assigned to the same set. For the first assigned edge, only the first of those conditions needs to be satisfied. This process is repeated until there is no more change. Next, the shortest-path-algorithm is executed for all node pairs within the matched artery edge sets and its longest paths is selected as the final result for one artery to avoid branching. This approach is hence similar to Shen et al. [2] without the trainable extension.

#### Metrics

Previous work used simple evaluation schemes such as classification into correct/incorrect [2] or accepted short artery segments (e.g., 10 mm anywhere in the A. basilaris [7]) which may be insufficient for high precision tasks. Hence, we suggest a finer evaluation framework with multiple subclasses listed in Table 1. First and most importantly, it is reported whether the whole vessel path was labeled correctly. Note that one needs to differentiate between vessels inside, leading to the CoW and those leading away from it, see Fig. 1. The vessels inside the CoW can be clearly defined by their bordering bifurcations while the rest of them only have one bordering bifurcation. Outgoing vessels, however, soon branch into multiple sub-arteries making it harder to define a single correct path. Hence, we evaluate only within a distance of up to 2 cm after the bifurcation which includes more than the important first segments of the A. media and posterior (M1 and P1). Since the incoming vessels, ICA and A. vertebralis, can be defined much more clearly, their whole segmented length will be assessed in the evaluation. The placement of the bifurcation is also reported as it influences the performance of the labeling. The category “$\le$ 5 mm” reports if a bifurcation is placed incorrectly, but within 5 mm from the real bifurcation, i.e., only slightly off but still located on two correct arteries.

## Results

### Bifurcations

The results are presented in Table 2, showing a labeling accuracy of 84.8%, which can be extended to 92.5% if slightly misplaced bifurcations are included as well, meaning that 7.5% of the bifurcations were not placed in the correct area. With 91% and 94%, the Vertebral and Posterior branchings are matched most reliably. Within a 5 mm tolerance, all bifurcations accuracies are over 90%. Without our consistent labeling design, the baseline does not focus on bifurcations (defined in the same manner as for our algorithm: classified as correct when all three arteries end at the same node), leading to lower accuracies for this secondary task with a mean of 26.8% (34.8% within 5 mm) which are thus not reported in detail.

### Vessel labeling

**Fig. 4:**
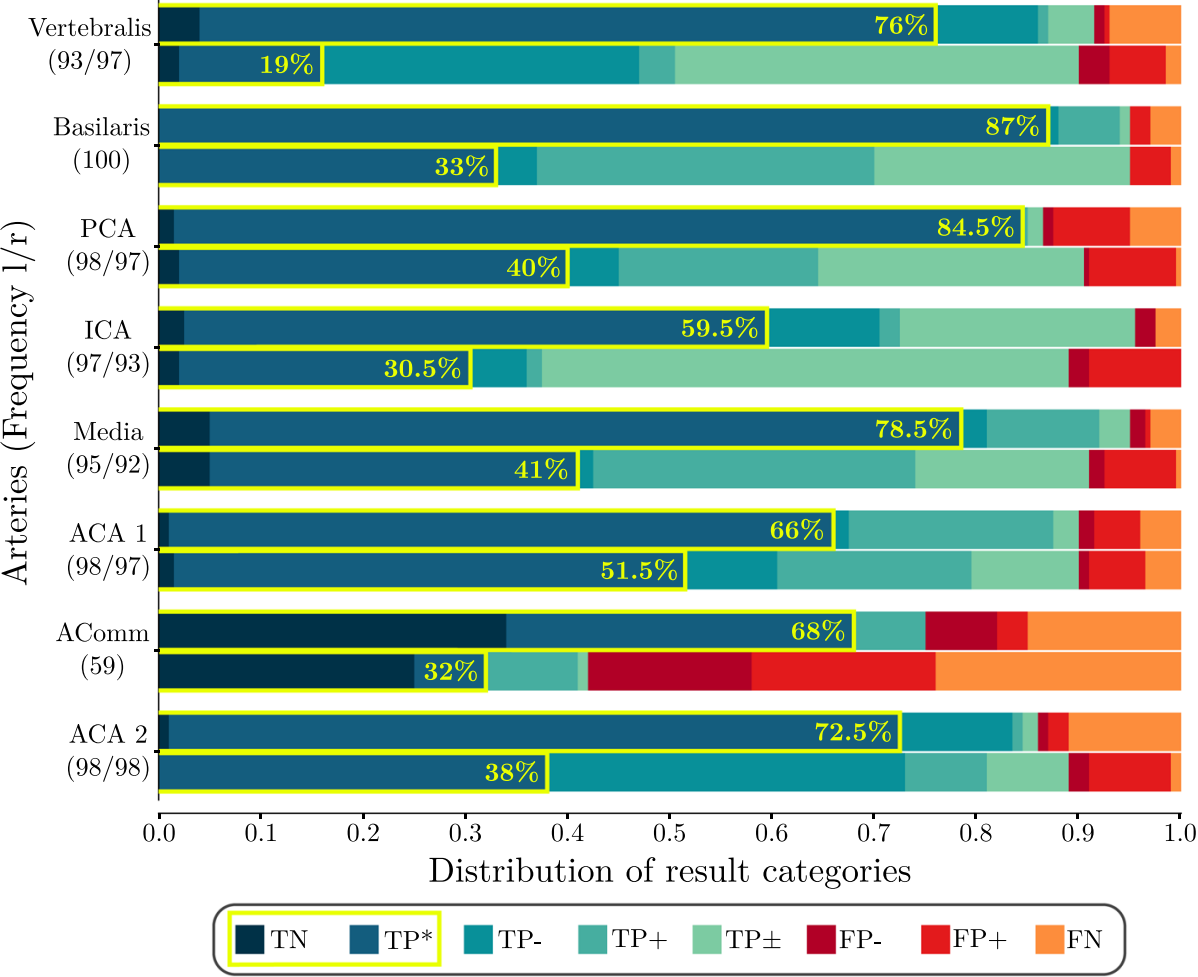
Results per artery for the proposed method (upper bar) and the baseline (lower bar). Yellow outline indicates fraction of perfectly labeled cases

**Fig. 5:**
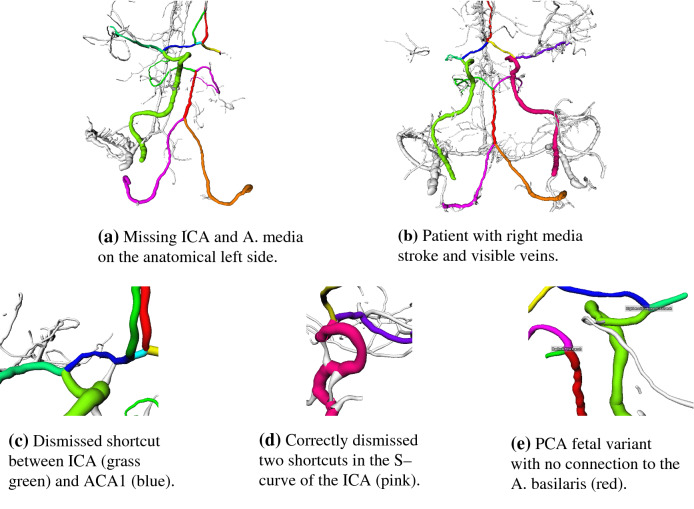
Labeled vessel graphs in row one and special cases in row 2. Each color represents a different vessel while white represents the unlabeled segments

The baseline and proposed method are evaluated manually using the model and CTA scan according to Table 1. Looking at the overall mean calculated from the values in Fig. 4, our proposed method achieves a detection rate ($\text {TP}_{\textsc {SUM}}$ and TN) of close to 90% on average over all vessels, with complete labeling in 73.5% of cases (TN and TP* only). Examples of complete labelings are presented in Fig. 5a, b. While the baseline approach also has a good overall detection rate of 86.6%, the fraction of completely labeled cases is only 35.5% on average.

The artery with the highest failure rate in both our method and the baseline is the small AComm with 25% incorrect estimations for our method and 55% for the baseline. Note that AComm is missing 41 times in our data sets which was detected correctly in 82.9% of the missing cases by our method. The lowest rates for complete estimation are observed for the long ICAs (e.g., 114 TP* vs. 186 $\text {TP}_{\textsc {SUM}}$). The problems presented in Fig. 2 were, however, typically resolved as demonstrated in Fig. 5c, d. Underestimation of the ICA is directly related to the overestimation for ACA 1 and A. media.

While A. basilaris and PCA L have high complete estimation rates (TN, TP*) with 87% and 89%, the rate for PCA R is significantly lower, caused by 9 patients with the fetal variant on the right side compared to 2 on the left side. In this variant, one PCA is not connected to the A. basilaris but originates from the ICA resulting in mislabeling and hence in only one correct prediction for the PCA R, see Fig. 5e. However, when a PCA was detected correctly it was also completely labeled in 97.8% (left) and 97.5% (right) of the cases.

Among the 100 test data sets, we encountered 66 patients with strokes or missing segments due to segmentation errors in critical areas, most often in the A. media. Summarized over both media arteries, 27 arteries were incomplete and 13 completely missing. Overall, the method could match 26 (22 complete) of the interrupted and recognized 10 of the missing ones as such, thus working in 90% of cases with an occluded A. media which matches the overall mean.

## Discussion

Our results demonstrate that inclusion of bifurcation matching improves the labeling of the artery paths. We modified the approach by Shen et al. [2] by using landmark registration and the shortest-path algorithm without the trainable part to construct a baseline. Even though it performs similarly for a rough detection of the arteries compared to the proposed approach, it leads to some kind of misestimation in almost 2/3 of all cases, rendering it infeasible for, e.g., straightening techniques. Furthermore, the labeling of the CoW may not be consistent as transitions between neighboring arteries are not enforced.

The problem of high FP and FN rates at the AComm (less relevant for stroke analysis) is caused by the length component of the score leading to overestimation of the short artery and hence defying the desired purpose here. In general, the anterior, especially ACA 2, is the hardest part of the vessel labeling [2] and, with a detection rate (TN and $\text {TP}_{\textsc {SUM}}$) of 86%, our algorithm is quite stable regarding missing A. communicans leading to only one ACA 2.

The fetal variant of the PCA is the most obvious failure case of the algorithm caused by the fixed consistent bifurcation condition of the method. In general the detection rate in all posterior arteries is robust as they are mostly straight vessels, with less complex geometry. Using the example of the clinically highly relevant A. media stroke case, we still achieve detection rates of 90% and can handle completely missing sub-trees as well as incomplete vessels.

We were able to show the benefit of our recursive bifurcation-based method when it comes to complete labeling, i.e., tracing completely correct from start to end node, which is essential for many applications. This method was developed on a very small set of five patients and generalized well over unseen data.

## Conclusion

In summary, we presented a robust algorithm for labeling the major brain vessels along the CoW in CTA scans of stroke patients. It is designed for artifact-prone cerebrovascular models derived from existing CTA segmentations, which can suffer from erroneous vessel paths. It is the first algorithm of its kind to be specifically evaluated on stroke patients. The method works on vessel graphs and recursively calculate scores for multiple paths. This score only relies on basic features such as smoothness at transitions, radius, length and distance to atlas landmarks. Maximizing these scores, six bifurcation points are identified along with 14 arteries around the CoW. This approach allows to obtain consistent and full labeling of the CoW. We show that while a purely atlas-distance driven method achieves similar vessel detection rates, it fails to provide complete labeling. The algorithm was developed on 5 and evaluated on 100 CTA data sets, including 66 cases with stroke or missing segments in the area around the CoW. Overall, we could find/detect arteries in 90% of the cases on average and were able to trace the full vessel path in 74%. A 90% detection accuracy is also achieved in cases with an A. media stroke.
